# On Aconite in Lichen and Prurigo

**Published:** 1851-10

**Authors:** 


*On Aconite in Lichen and Prurigo.* By M. CAZENAVE.—Believing that, in papular affections of the skin, the essential thing is to subdue hyperæsthesia, of which the eruption is but the consequence, M. Cazenave has been long in the habit of employing the extract of aconite with great success. The intense and irresistible itching rapidly diminishes, and afterwards ceases. He divides fifteen grains into forty pills and gives one or two night and morning.—*British and For. Med. Chir. Rev., from Bullet. de Thérap.*

